# Anti-Tumor Potential of Selective L-Type Amino Acid Transporter (LAT1) Inhibitor, JPH203, in Patient-Derived Canine Bladder Cancer Organoids

**DOI:** 10.3390/ijms27177681

**Published:** 2026-08-27

**Authors:** Ting-Wei Yu, Minami Kawahashi, Fumiya Goda, Ami Kanaya, Mohamed Elbadawy, Hitoshi Endou, Shoichiro Ide, Hideyuki Yamawaki, Masahiro Kaneda, Amira Abugomaa, Tsuyoshi Uchide, Tatsuya Usui, Kazuaki Sasaki

**Affiliations:** 1 Laboratory of Veterinary Pharmacology, Department of Veterinary Medicine, Faculty of Agriculture, Tokyo University of Agriculture and Technology, 3-5-8 Saiwai-cho, Fuchu 183-8509, Japan; 2 Laboratory of Veterinary Molecular Pathology and Therapeutics, Faculty of Agriculture, Tokyo University of Agriculture and Technology, 3-5-8 Saiwai-cho, Fuchu 183-8509, Japan; 3 Department of Pharmacology, Faculty of Veterinary Medicine, Benha University, Moshtohor, Toukh 13736, Egypt; 4 Codec Chemical Co., Ltd., 1-1-4, Kanda, Ogawa-Machi, Chiyoda-ku 101-0052, Japan; 5 Laboratory of Veterinary Pharmacology, School of Veterinary Medicine, Kitasato University, 35-1 Higashi 23 ban-cho, Towada 034-8628, Japan; 6 Laboratory of Veterinary Anatomy, Department of Veterinary Medicine, Faculty of Agriculture, Tokyo University of Agriculture and Technology, 3-5-8 Saiwai-cho, Fuchu 183-8509, Japan; 7 Faculty of Veterinary Medicine, Mansoura University, Mansoura 35516, Egypt

**Keywords:** canine, urothelial carcinoma, JPH203, L-type amino acid transporter, mTOR pathway

## Abstract

L-type amino acid transporter 1 (LAT1), which controls the neutral amino acid uptake across the cell membranes, has been frequently overexpressed in various human cancers. Overexpression of LAT1 is associated with higher proliferation and shorter patient survival in tumors. Here, upregulated LAT1 expression was observed in patient-derived canine bladder cancers (BCs), and a small-molecule LAT1 inhibitor (JPH203) successfully reduced BC tumor growth both in vitro and in vivo. Patient-derived cancer cells were collected from dogs with naturally occurring tumors at veterinary clinics in Japan, and the 2.5D cancer organoid culture system was generated following our previous studies. Compared with other canine cancers or normal bladder cells, the increased LAT1 expression was observed in 2 strains of BC organoids. JPH203 inhibited boronophenylalanine (BPA) uptake activity by more than 90% and suppressed cell proliferation dose-dependently. Deprivation of BPA uptake activity by JPH203 also regulated the phosphorylation of mTOR-related proteins. Furthermore, intraperitoneal administration of JPH203 decreased the growth and tumor weight of BC organoid-derived xenograft in immunodeficient mice with an induction of apoptosis and a decrease in LAT1 expression compared to vehicle-administered mice. Therefore, the present study demonstrates that JPH203 exerts anti-tumor effects in canine BC through modulation of the mTOR signaling pathway and supports further investigation of LAT1-targeted therapy for canine BC.

## 1. Introduction

Cancer cells can modulate nutrient uptake to support rapid cell proliferation. Among these nutrients, glucose consumption is the most commonly used biomarker in cancer. However, it is not specific to cancer. On the other hand, amino acid hypermetabolism has been exhibited as a potential biomarker of cancer due to lower expression in normal cells, suggesting a higher tumor-specificity and fewer side effects [1,2,3]. L-type amino acid transporter (LAT) family, including four types of membrane transport proteins (LAT1-4), is known to be responsible for the delivery of amino acids within and between cells and plays an important role in cell proliferation by controlling amino acid transport. LAT1 (SLC7A5) has been observed to be overexpressed in a variety of human cancers, such as breast cancer [4,5], prostate cancer [6], bladder cancer [7,8], brain tumors [9], skin tumors [10], and digestive organ cancers [11,12,13]. Importantly, LAT1 showed deficient expression in normal tissues. Therefore, LAT1 has been used as a molecular target for cancer diagnosis and treatment in humans [13,14,15,16]. Recently, (2S)-2-amino-3-[4-[(5-amino-2-phenyl-1,3-benzoxazole-7-yl) methoxy]-3,5-dichlorophenyl] propanoic acid (JPH203), a selective inhibitor of LAT1, was designed and synthesized by Endou et al. [17]. and has passed phase I study in patients with advanced solid tumors [18]. The antitumor effects of JPH203 have been investigated in several types of human cancers, including colorectal cancer, osteosarcoma, and cholangiocarcinoma, both in vitro and in vivo [2]. JPH203 demonstrated excellent potential for inhibiting cell proliferation and suppressing tumor growth in pre-clinical evaluations.

Besides in humans, LAT1 has also been revealed at a high level in canine naturally occurring cancers such as canine malignant melanoma (ML) [10], canine mammary tumor (MT) [19], canine intracranial tumor, and canine hepatocellular carcinoma (HCC) [20] compared to normal tissues. Moreover, Fukumoto et al. found a significantly upregulated expression of LAT1 in ML patients with distant metastasis compared to those without metastasis [10]. In addition, invasive malignant mammary tumors predominantly expressed LAT1 compared with non-invasive mammary tumors [19]. Based on these results, LAT1 expression showed promise as a clinical marker for predicting cancer behavior and prognosis. However, there are no complete in vitro and in vivo therapeutic results of the LAT1 inhibitor, JPH203, against canine malignant tumors.

In the previous studies, we established a 2.5-dimensional (2.5D) organoid culture model, which could maintain the expression of specific markers with their original cancer tissues and exhibited tumorigenesis in vivo, by using companion animal cancer tissues or urine [21]. Herein, LAT1 expression was characterized in five different patient-derived 2.5D canine cancer organoids. The 2.5D canine bladder cancer (BC) organoids, which exhibited the highest LAT1 gene expression among five types of cancers, were used to examine in vitro therapeutic effects of JPH203 treatment. Also, the influence of JPH203 on protein translation was examined. Furthermore, an in vivo study of JPH203 treatment was carried out on xenografted BC 2.5D organoids in immunodeficient mice.

## 2. Results

### 2.1. Evaluation of LAT1 Expression in Patient-Derived Canine Tumor Organoids

**Figure 1:**
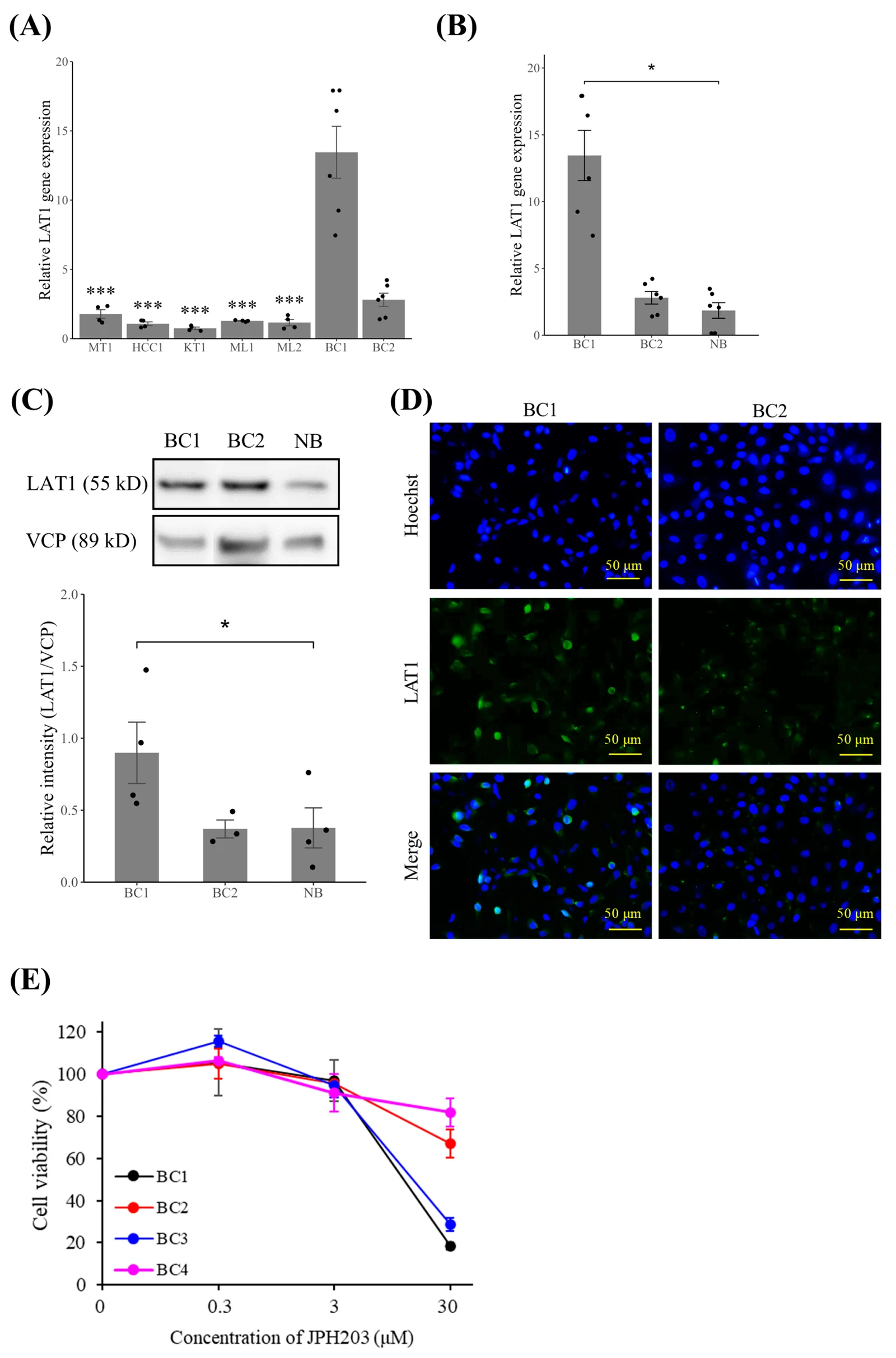
LAT expression in various types of canine cancer cells. mRNA expression was evaluated by real-time PCR ((**A**), n = 3–5). Results were expressed as mean ± S.E.M, *** *p* < 0.001 vs. BC1. Relative quantification was normalized to glyceraldehyde-3-phosphate dehydrogenase (GAPDH). Abbreviations: MT, mammary tumor; HCC, hepatocellular carcinoma; KT, kidney tumor; ML, melanoma; BC, bladder cancer; NB, normal bladder. Comparison of LAT expression in canine BC cells. mRNA expression was evaluated by real-time PCR ((**B**), n = 3–5). Results were expressed as mean ± S.E.M. * *p* < 0.05 vs. BC1. Relative quantification was normalized by GAPDH. Protein expression was evaluated by Western blotting analysis ((**C**), n = 3) and immunofluorescence staining ((**D**), n = 3). Results were expressed as mean ± S.E.M.VCP was used as a protein-loading control. * *p* < 0.05 vs. BC1. Scale bar: 50 µm. Cell viability of BC1, BC2, BC3, or BC4 after JPH203 treatment for 72 h (**E**). Results were expressed as mean ± S.D. (n = 3). The sample size varied among the experimental groups depending on the number of available samples; therefore, n = 3–5 indicates that three to five samples were analyzed per group.

LAT1 gene expression was evaluated in patient-derived 2.5D canine tumor organoids, including mammary adenocarcinoma (MT), hepatocellular carcinoma (HCC), renal cell carcinoma (KT), melanoma (ML), and urothelial carcinoma (BC), by comparative CT method. A significantly higher LAT1 expression was observed in canine BC organoids (specifically BC1) compared with other tumor organoids (Figure 1A). Compared with normal bladder (NB) organoids, BC1 showed significantly higher LAT1 (SLC7A5) mRNA expression, as determined by quantitative RT-PCR (Figure 1B), and higher LAT1 protein expression, as determined by Western blot analysis (Figure 1C). Consistently, LAT1 expression was also detected in BC organoids by immunofluorescence staining (Figure 1D). The percentage of LAT1-positive cells was 53.6% in BC1 organoids and 36.8% in BC2 organoids. Next, the inhibition of cell viability by JPH203 was evaluated in 4 different strains of BC organoids, and all of them showed a concentration-dependent response (Figure 1E). 30 µM of JPH203 treatment revealed an extremely high cell inhibition rate against BC1 (81.5%) and BC3 (71.2%). By contrast, BC2 and BC4 showed resistance to JPH203 treatment. Furthermore, mitoxantrone and vinblastine, two chemotherapeutic agents commonly used for the treatment of canine bladder cancer, were employed to evaluate the potential of combination therapy with JPH203. Treatment with JPH203 (10 μM) in combination with vinblastine (0.01–10 nM) significantly reduced BC1 cell viability at a vinblastine concentration of 1 μM. In contrast, no synergistic effect was observed when JPH203 was combined with mitoxantrone (1–100 ng/mL) (Figure S1).

### 2.2. Inhibition of BPA Uptake Activity in BC Organoids by JPH203

**Figure 2:**
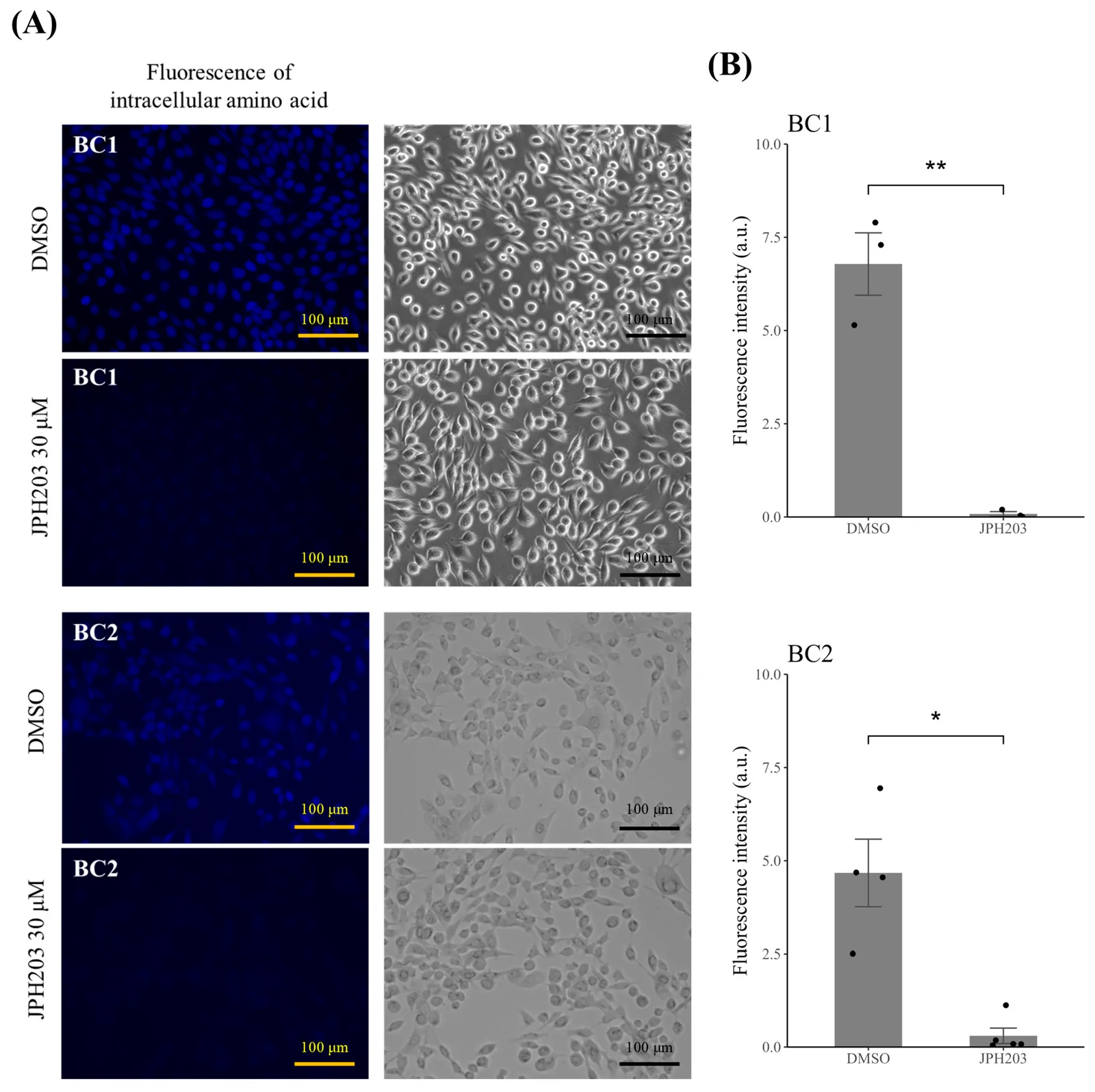
The inhibition of amino acid transporter was detected by boronophenylalanine (BPA) uptake activity assay in bladder cancer cells. Fluorescence was detected after culturing BC1 or BC2 organoids with amino acid analogs (boronophenylalanine) and the membrane-permeable fluorescent probe, followed by JPH203 or vehicle treatment (**A**). The blue fluorescence represents the signal generated by the BPA uptake assay. The quantification of fluorescence intensity was normalized by cell number in each image ((**B**), n = 3). Results were expressed as mean ± S.E.M. * *p* < 0.05, ** *p* < 0.01 vs. Control. Scale bar: 100 µm.

The effects of the LAT1 inhibitor JPH203 on amino acid uptake capacity were investigated in patient-derived BC 2.5D organoids. JPH203 treatment significantly suppressed BPA uptake capacity in both BC1 and BC2 organoids compared with vehicle treatment (Figure 2).

### 2.3. Regulation of the mTOR Signaling Pathway in BC Organoids by JPH203

**Figure 3:**
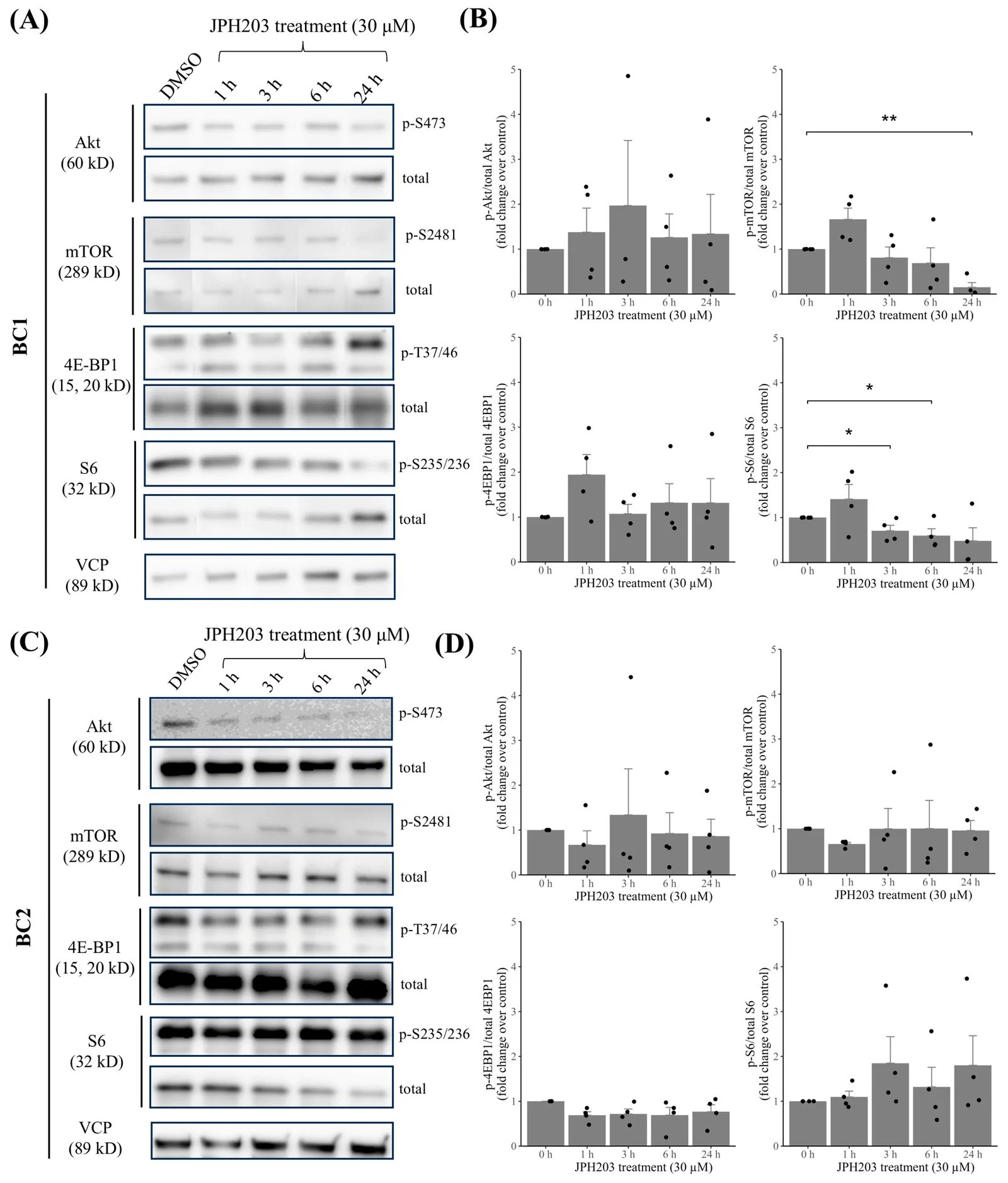
The protein expressions of Akt, p-Akt, mTOR, p-mTOR, 4E-BP1, p-4E-BP1, ribosome (S6), and p-S6 in 2.5D BC1 or BC2 organoids following JPH203 treatment were determined by Western blot analysis ((**A**,**C**), n = 6 for BC1 and n = 4 for BC2). VCP was used as a protein-loading control. (**B**,**D**) The phosphorylation levels of Akt, mTOR, 4E-BP1, and S6 were quantified as the ratios of phosphorylated to corresponding total protein (p-Akt/Akt, p-mTOR/mTOR, p-4E-BP1/4E-BP1, and p-S6/S6) and are presented relative to those at 0 h. Results were expressed as mean ± S.E.M. * *p* < 0.05, ** *p* < 0.01 vs. 0 h.

The effects of JPH203 on the mTOR signaling pathway in BC1 and BC2 organoids were examined (Figure 3). Among mTOR signal-related proteins (Akt, mTOR, 4E-BP1, and S6), phosphorylation of mTOR was significantly decreased in BC1 24 h after JPH203 treatment (Figure 3A,B). Phosphorylation of ribosomal protein S6 was also significantly decreased on BC1 at 3 and 6 h after treatment. Phosphorylation of Akt and 4E-BP1 was not significantly changed on BC1 during 24 h JPH203 treatment. On the other hand, no significant change was found in the phosphorylation level of these four proteins related to the mTOR signaling pathway on BC2 during 24 h JPH203 treatment (Figure 3C,D). In addition, JPH203 treatment reduced cyclin D1 protein levels in BC organoids (Figure S2).

### 2.4. In Vivo Tumor Growth Inhibitory Effect of JPH203 in Xenograft BC Mouse Model

**Figure 4:**
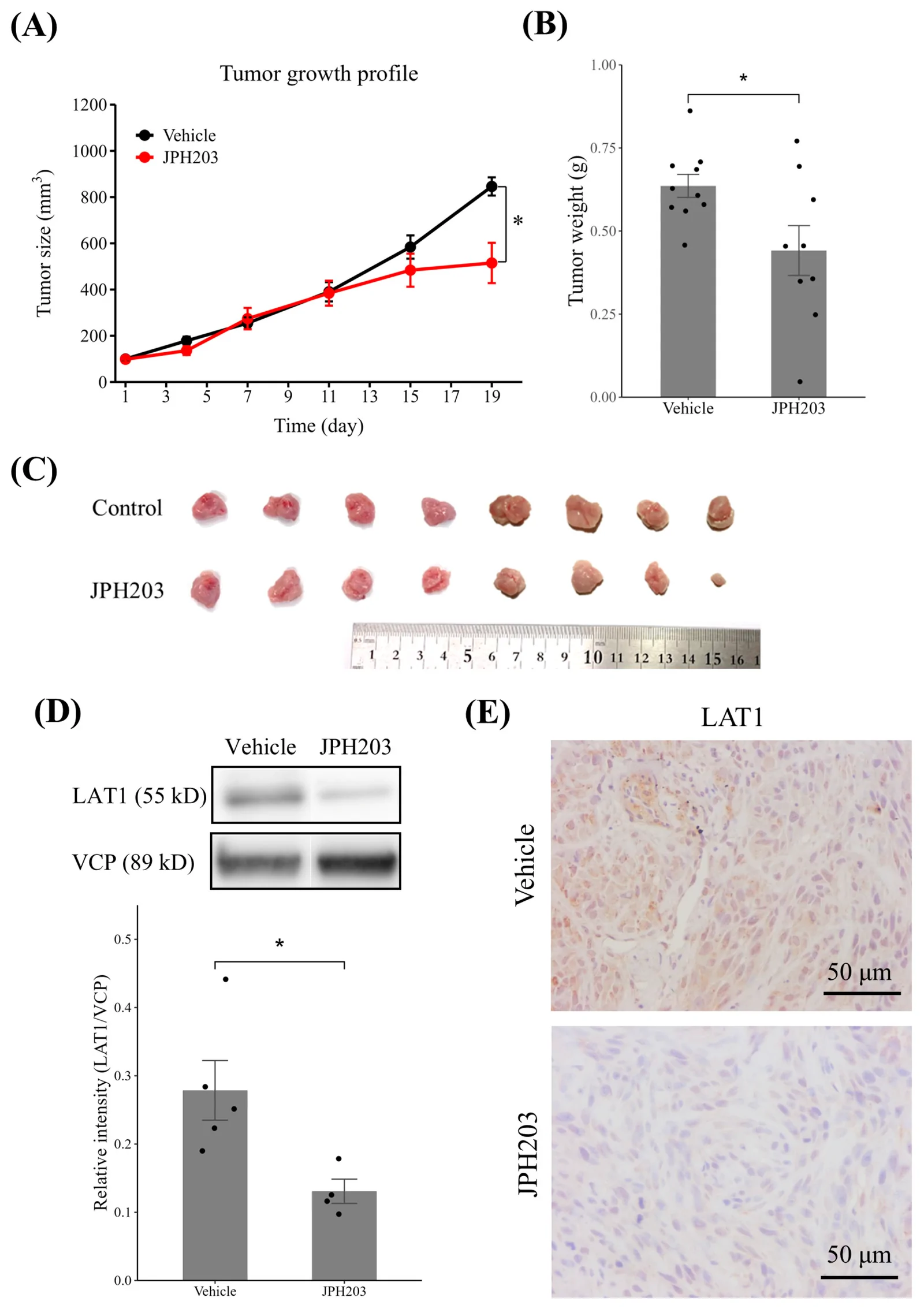
Tumor growth inhibitory effect of JPH203 against canine BC1 organoids-bearing immunodeficient mice. The tumor size was measured two times a week for 19 days ((**A**), n = 10). Results were expressed as mean ± S.E.M. * *p* < 0.05 vs. Vehicle. Averaged tumor weight (**B**) and photographs (**C**) of tumors were evaluated from the mice sacrificed at day 19 after JPH203 treatment (n = 10). Results were expressed as mean ± S.E.M. * *p* < 0.05 vs. Vehicle. LAT1 expression in harvested tumor tissues from the mice sacrificed at day 19 after JPH203 treatment was examined by Western blotting analysis ((**D**), n = 4) and immunohistochemistry staining ((**E**), n = 3). VCP was used as a protein-loading control. Results were expressed as mean ± S.E.M. * *p* < 0.05 vs. Vehicle. Scale bar: 50 µm.

Finally, the anti-tumor activity of JPH203 was examined on BC organoid-implanted immunodeficient mice. JPH203 was administered intraperitoneally five times per week at a dose of 50 mg/kg. The tumor growth was inhibited by JPH203 15 days after administration, and a significantly decreased mean tumor size was observed on day 19 compared to control mice (Figure 4A). The individual tumor growth curves are shown in Figure S3. By day 19 post-treatment, the treated mice lost approximately 15% of their body weight compared to the control group (Figure S4). As JPH203 may restrict the uptake of essential amino acids, the weight loss is probably due to amino acid deprivation and reduced protein synthesis rather than non-specific toxicity. No mice showed signs of severe pain, fever, or other toxicity, including lethargy, piloerection, or reduced activity.

**Figure 5:**
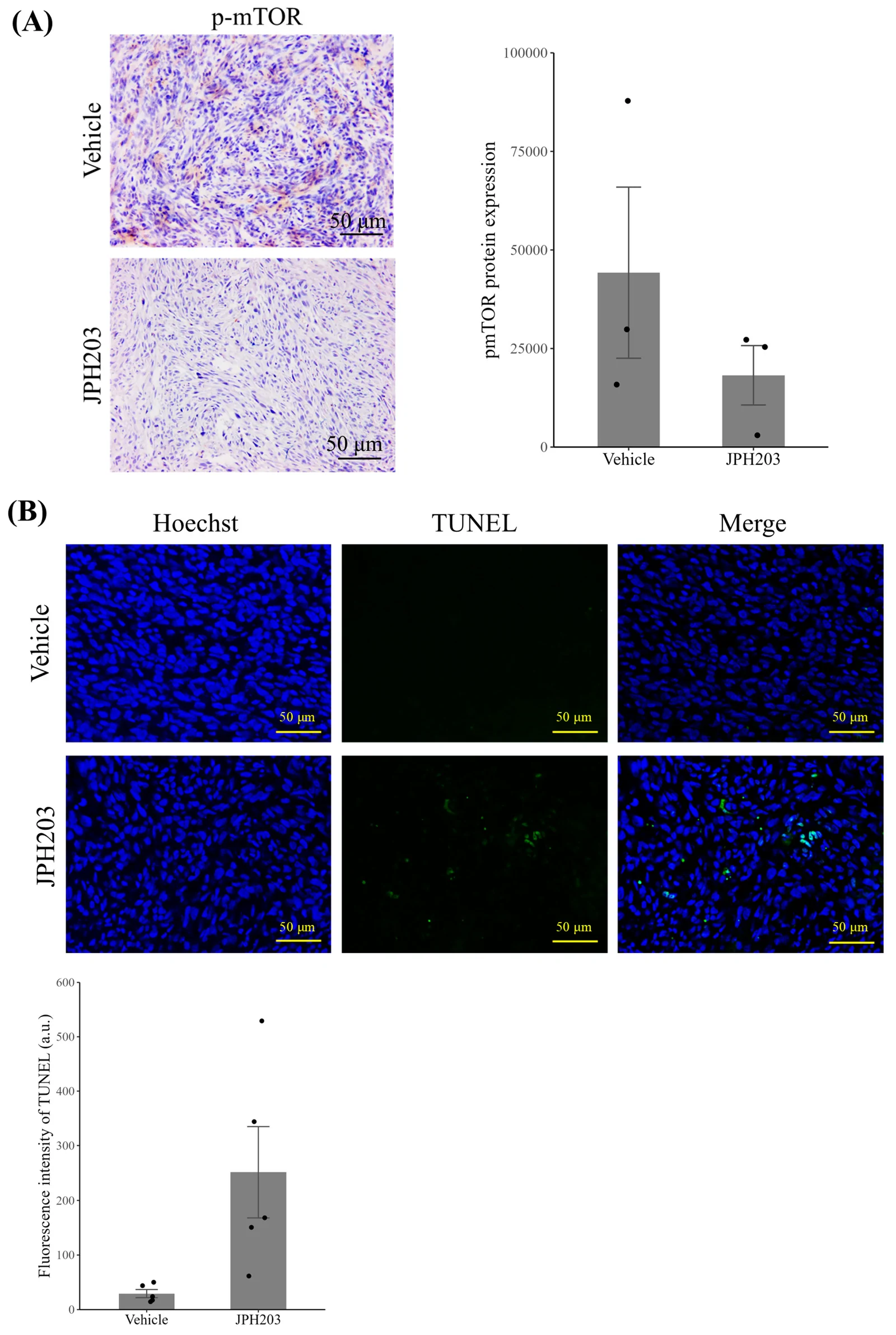
Immunohistochemistry staining of phospho-mTOR in harvested tumor tissues from the mice sacrificed at day 19 after JPH203 treatment. The quantification of immunohistochemistry intensity was normalized by cell number in each image ((**A**), n = 5). Results were expressed as mean ± S.E.M. TUNEL staining in harvested tumor tissues from the mice sacrificed at day 19 after JPH203 treatment. The quantification of TUNEL intensity was normalized by cell number in each image ((**B**), n = 5). Scale bar: 50 µm.

After the third JPH203 administration cycle, mice were sacrificed, and tumors were collected for further experiments. As shown in Figure 4B,C, the mean weight of tumors was significantly decreased after JPH203 treatment, although some showed a diminished response to JPH203 treatment. Moreover, LAT1 expression was examined on xenografted BC tumors after JPH203 treatment by Western blotting and immunohistochemical staining. As shown in Figure 4D,E, LAT1 protein expression was significantly decreased after JPH203 treatment compared to the control group, indicating a change in LAT1 expression after LAT1-targeted treatment. Next, the regulation of the mTOR signal was also evaluated in the xenografted BC tumor tissues. JPH203-administered tumor tissues revealed lower expression of phosphorylation of mTOR (Figure 5A). Furthermore, TUNEL assay was conducted in the xenografted tumor tissues to evaluate apoptotic cells after JPH203 administration. As shown in Figure 5B, some apoptotic cells were found in JPH203-administered tumor tissues, which were rarely found in the control group.

## 3. Discussion

Canine BC, also called urothelial carcinoma, comprises 1.5 to 2.0% of all canine cancers, and more than 90% of BC in dogs are intermediate- to high-grade invasive cancers [22]. Most bladder cancers in dogs are challenging to excise surgically due to the trigonal location of the tumor, and metastasis often happens as a result of delayed diagnosis. Systemic medical therapies using chemotherapy agents and nonsteroidal anti-inflammatory drugs (NSAID) are mainly conducted in human and canine BC treatment [23,24]. However, the responses are poor, with median survival of about 4–6 months after cisplatin, carboplatin, or piroxicam treatment. Therefore, new therapeutic approaches are necessary to break the deadlock in canine BC treatment.

High LAT1 expression has been reported in several types of canine tumors, including mammary tumors [19], malignant melanoma [10], and hepatocellular carcinoma [20]. In the present study, we for the first time proposed LAT1 as a cancer biomarker in canine BC and examined the therapeutic potential of the LAT1 inhibitor, JPH203, on patient-derived canine BC organoids. Compared to normal bladder organoids, BC organoids showed higher LAT1 expression, and the in vitro cell growth inhibitory effect of JPH203 against BC organoids showed a positive correlation with LAT1 level, suggesting JPH203 selectively inhibited cell proliferation in LAT1-overexpressing cancer cells. Furthermore, JPH203 suppressed the growth of cancer cells in SCID mouse-transplanted BC organoids, particularly those that overexpressed LAT1. This contributed to a decrease in the overall amount of LAT1 protein in tumor tissues. Hence, the blockade of LAT1 by JPH203 may represent a novel therapeutic approach in BC treatment.

In previous studies, LAT1-mediated L-leucine uptake has been reported to affect cancer cell proliferation through the mTOR signaling pathway in human bladder cancer [7]. mTOR is a member of the phosphoinositide-3-kinase related kinase (PI3K) family which consists of two major complexes, mTORC1 and mTORC2. mTORC1 regulates protein translation by activating S6 kinase and repressing elF-4E-binding protein (4E-BP1), while it is activated by both PI3K/Akt signaling and amino acids (especially leucine, arginine, and glutamine) [25,26]. In the previous report, Nishikubo et al. found that the phosphorylation of p70S6K, S6 ribosomal protein, and 4E-BP1 was decreased by JPH203 treatment in four human pancreatic cancer cell lines, indicating the reduced kinase activity of mTORC1 [27]. Maimaiti et al. also reported that JPH203 treatment inhibited phosphorylation of Akt, p70S6K, and 4E-BP1 in the human BC cell line [7]. In the present study, the regulation of the mTOR pathway through the LAT1 inhibitor, JPH203, was also demonstrated in vitro and in vivo. Although the phosphorylation level of Akt and 4E-BP1 was not changed obviously in BC organoids, the phosphorylation of mTOR and S6 was significantly inhibited after JPH203 treatment, as shown by Western blotting analysis in vitro (Figure 3B).

Further, the decreased phospho-mTOR protein expression was found in JPH203-treated SCID mouse-transplanted BC organoids (Figure 5A). These findings suggest that chronic inhibition of LAT1 deprives cancer cells of essential amino acids, thereby suppressing the mTOR signal [28], a major amino acid-sensing pathway, which may subsequently downregulate LAT1 expression and ultimately inhibit cell proliferation in canine BCs. Although the antitumor effect of JPH203 in vivo was relatively modest, tumor cells may compensate by using alternative nutrient-acquisition pathways or metabolic rewiring. Nevertheless, these results indicate that LAT1-mediated BPA uptake plays a functionally important role in mTOR activation and tumor growth regulation, even though blockade of this pathway alone may be insufficient to induce profound tumor regression in vivo. Consistent with this notion, LAT1 blockade by JPH203 increased the proportion of TUNEL-positive cells in mouse-transplanted BC organoids (Figure 5B), indicating that JPH203 suppresses tumor growth, at least in part, by inducing apoptotic cell death. Consistent results were also observed in vitro, where JPH203 treatment reduced cyclin D1 protein levels in BC organoids (Figure S2), in agreement with findings from previous reports [16,28].

Because JPH203 is a competitive inhibitor of LAT1, relatively high concentrations may be required under conventional in vitro culture conditions, where culture media contain supraphysiological concentrations of amino acids that compete with JPH203 for LAT1-mediated transport. Consistent with this, recent studies investigating the anti-tumor effects of JPH203 have used concentrations ranging from 30 to 100 μM in human cancer cells [29,30,31]. Although these concentrations exceed the plasma concentrations currently achievable in clinical studies and the potential contribution of off-target effects cannot be completely excluded, treatment with 30 μM JPH203 in the present study significantly inhibited BPA uptake and suppressed mTOR signaling, consistent with the expected biological consequences of LAT1 inhibition. Therefore, the present findings should be interpreted as mechanistic evidence supporting the anti-tumor effects of LAT1 inhibition under in vitro conditions rather than as a direct prediction of clinical efficacy.

Although LAT1 primarily functions at the plasma membrane, previous studies have reported that LAT1 is also localized in intracellular compartments during protein biosynthesis and intracellular trafficking [4,15,32]. In addition, although the specificity of the LAT1 antibody for canine immunohistochemistry was not independently validated, LAT1 expression and activity were consistently supported by quantitative RT-PCR, Western blotting, immunofluorescence, BPA uptake, and mTOR signaling analyses. Although the pharmacological, molecular, functional, and in vivo findings support the involvement of LAT1 in the effects of JPH203, genetic validation was not performed in the present study. Future studies using SLC7A5 knockdown or knockout approaches will be required to further establish the direct involvement of LAT1.

The pharmacokinetics of JPH203 have been evaluated in humans [33], where the maximum plasma concentration reaches approximately 3 μM following administration at a maximal tolerated dose of 60 mg/m^2^. However, pharmacokinetic and safety data for JPH203 in dogs are currently lacking. Since both in vitro and in vivo results in this study indicated that a relatively higher concentration of JPH203 was required to achieve optimal therapeutic efficacy, a dose-dependent pharmacokinetic and safety study in dogs will be necessary to support its future clinical application in veterinary oncology. In this context, the present study provides the first evidence that JPH203 exerts anti-tumor effects in canine BC organoids and supports further investigation of LAT1-targeted therapy for canine BC.

## 4. Materials and Methods

### 4.1. Cell Line Validation

No established or commercially available cell lines were used in this study. All experiments were performed using patient-derived canine bladder cancer organoids and normal bladder organoids established from clinical samples. Therefore, cell line authentication was not applicable.

### 4.2. Chemicals and Antibodies

LAT1 inhibitor, JPH203, was kindly provided by Codec Chemical Co., Ltd. (Tokyo, Japan). PrestoBlue^TM^ Cell Viability Reagent was purchased from Thermo Fisher Scientific Inc. (Waltham, MA, USA), and Amino Acid Uptake Assay Kit was purchased from Dojindo Laboratories Co., Ltd. (Kumamoto, Japan). Tween^®^ 20 was purchased from Sigma-Aldrich (St. Louis, MO, USA) and dimethyl sulfoxide (DMSO) was purchased from Wako Pure Chemical Corporation (Osaka, Japan). JPH203 was prepared as a 10 mM stock solution in DMSO. The final concentration of DMSO was 0.3% (*v*/*v*) at the highest JPH203 concentration (30 μM), and the corresponding vehicle control contained the same concentration of DMSO.

For organoid culture, Advanced Dulbecco’s Modified Eagle’s Medium (DMEM)/F12 (Thermo Fisher Scientific Inc., Waltham, MA, USA) was supplemented with 10 mM 4-(2-hydroxyethyl)-1-piperazineethanesulfonic acid (HEPES; Wako Pure Chemical Corporation, Osaka, Japan), 1% GlutaMAX (Thermo Fisher Scientific Inc., Waltham, MA, USA), 10 mM nicotinamide (Sigma-Aldrich, St. Louis, MO, USA), 1 mM N-acetyl-L-cysteine (Sigma-Aldrich, St. Louis, MO, USA), 0.5 μM A83-01 (Adooq Bioscience, Irvine, CA, USA), 1% penicillin-streptomycin (Wako Pure Chemical Corporation, Osaka, Japan), and 5% fetal bovine serum (FBS; Thermo Fisher Scientific Inc., Waltham, MA, USA).

SLC7A5 (LAT1) antibody (catalog No. orb158412) was purchased from Biorbyt Ltd. (Cambridge, UK). mTOR antibody (catalog No. 2972), Phospho-mTOR (Ser2481) antibody (catalog No. 2974), Akt antibody (catalog No. 9272), Phospho-Akt (Ser473) antibody (catalog No. 4060), 4E-BP1 (53H11) antibody (catalog No. 9644), Phospho-4E-BP1 (Thr37/46) (236B4) antibody (catalog No. 2855), S6 Ribosomal Protein (54D2) antibody (catalog No. 2317), and Phospho-S6 Ribosomal Protein (Ser235/236) (D57.2.2E) antibody (catalog No. 4858) were purchased from Cell Signaling Technology Inc. (Danvers, MA, USA). VCP antibody (catalog No. GTX113030) was purchased from GeneTex Inc. (Irvine, CA, USA). The secondary antibodies were as follows: Goat Anti-Rabbit IgG H&L (Alexa Fluor^®^ 488) (catalog No. ab150077, Abcam, Cambridge, UK) and Horseradish peroxidase (HRP)-conjugated anti-rabbit IgG (catalog No. 10004301, Cayman Chemical, Ann Arbor, MI, USA). Dako Envision+ dual Link System-HRP (catalog No. K4061) was purchased from Agilent Technologies Inc., Santa Clara, CA, USA. Polymerase chain reaction (PCR) primers were from FASMAC Co. Ltd. (Atsugi, Japan). Hoechst 33258 solution (catalog No. 19173-41, Nacalai Tesque Inc., Kyoto, Japan) was used for nucleus staining.

### 4.3. Organoid Preparation and Culture

Between 2019 and 2022, canine tumor tissues or urine samples were collected from animal clinics in Japan, and the detailed information is listed in Table 1. Written informed consent for the current study was obtained from all dog owners, and the study was conducted under the direction of the Institutional Animal Care and Use Committee of Tokyo University of Agriculture and Technology (Approval No. 0020007; approval date: 8 May 2023). The samples were transferred immediately to our laboratory in a cooled shipping medium. The 2.5D tumor or normal organoids were generated, passaged, and processed as described in our previous study [21].

### 4.4. Quantitative Real-Time Reverse Transcription Polymerase Chain Reaction Assay

Total RNA was extracted from 2.5D organoids using FavorPrep Tissue Total RNA Mini Kit (FAVORGEN, Ping-Tung, Taiwan) following the manufacturer’s guidelines. The extracted total RNA samples were used for reverse transcription to generate complementary DNA (cDNA) by ReverTra Ace^TM^ qPCR RT Master Mix (TOYOBO Co., Ltd., Osaka, Japan). The quantitative real-time PCR was conducted using a QuantiTect SYBR I Kit (QIAGEN N.V., Venlo, The Netherlands) and a StepOne^TM^ Real-Time PCR System (Applied Biosystems, Waltham, MA, USA). The $\Delta \Delta C_{q}$ method was followed to quantify data using glyceraldehyde-3-phosphate dehydrogenase (GAPDH) as an endogenous control. Relative gene expression was calculated using the 2^−ΔΔCt^ method and presented as fold change. Statistical analyses were performed using ^ΔΔCt^ values rather than fold change values. Specific canine LAT1 primers used in this study were described as follows:

Forward: GCTGTGGACTTTGGGACCTA

Reversed: CTGGAGAAGGCGTAAAGCAG

### 4.5. Immunofluorescence Staining of LAT1 Expression

Immunofluorescence staining of BC 2.5D organoids was conducted as described before [21]. Briefly, the BC organoid cells (2 × 10^5^ cells/well) were seeded in a 6-well plate with coverslips. After 70% confluent conditions, the cells were fixed with 4% paraformaldehyde (PFA) solution for 30 min at room temperature (RT). After washing with PBS, the cells were blocked by 1.5% normal goat serum for 30 min. Later, the cells were treated with SLC7A5 (LAT1) antibody (1:200) for 120 min at RT. Subsequently, the secondary antibodies, Goat Anti-Rabbit IgG H&L (1:500) and Hoechst 33258 for nucleus staining, were treated, followed by a PBS washing process. After one h of reaction at RT in dark conditions, the expressions were examined using a microscope (BX52-DP72, OLYMPUS, Tokyo, Japan).

### 4.6. Western Blotting

The expression of LAT1 and the signal pathway-related proteins was examined by Western blot analysis following the standard guidelines. The protein samples from each cell were extracted by CelLytic™ M (Sigma-Aldrich, St. Louis, MO, USA). Equal protein amounts (10 μg) were loaded and separated by sodium dodecyl sulfate-polyacrylamide gel electrophoresis (SDS-PAGE; Mini-PROTEAN TGX Gels, Bio-Rad Laboratories, Inc., Hercules, CA, USA) and then transferred to PVDF membrane (Clear Blot P+ membrane, ATTO CORPORATION, Tokyo, Japan). Following protein blocking by skimmed milk or bovine serum albumin, the membranes were incubated with primary antibodies (SLC7A5, 1:500; mTOR, 1:500; p-mTOR, 1:500; Akt, 1:500; p-Akt, 1:500; 4E-BP1, 1:500; p-4E-BP1, 1:500; S6 Rib, 1:500; p-S6 Rib, 1:500; and VCP, 1:500) at 4 °C overnight. After washing, the secondary antibody (HRP-conjugated anti-rabbit IgG, 1:1000) was applied for 1 h at RT. Images were acquired using a LAS-3000 image analyzer (Fujifilm, Tokyo, Japan) and quantified using ImageJ software (National Institutes of Health, Bethesda, MD, USA; https://imagej.nih.gov/ij/, accessed on 27 August 2026). VCP was used as a loading control because of its abundant and relatively stable expression and its previous use as a loading control in canine bladder cancer organoids [34].

### 4.7. Cell Viability Assay of 2.5D BC Organoids

Cell viability assay of BC 2.5D organoids was conducted as described previously [21]. Briefly, BC organoid cells were seeded in a 96-well plate at a density of 1 × 10^3^ cells/well and incubated overnight. The cells were then treated with various concentrations of the LAT1 inhibitor, JPH203, for 72 h. Cell viabilities were evaluated with PrestoBlueTM Cell Viability Reagent (excitation wavelength: 560 nm; emission wavelength: 590 nm). The fluorescence intensity was measured by a microplate reader (TECAN Group Ltd., Männedorf, Switzerland).

### 4.8. Boronophenylalanine (BPA) Uptake Activity Assay

Amino acid transport activity in BC 2.5D organoids was quantified using a BPA uptake activity assay kit (Cat. No. UP04, Dojindo Laboratories, Kumamoto, Japan) following the manufacturer’s guidelines. Briefly, BC organoids were seeded in a 24-well plate at a density of 1 × 10^4^ cells/well and incubated overnight. The cells were washed three times with a 0.1% Glucose/PBS solution and then treated with 30 µM of JPH203 or DMSO (as a control) at 37 °C for 5 min. After removing the solution, organoids were fed a Boronophenylalanine (BPA) solution containing JPH203 or DMSO and incubated at 37 °C for an additional 5 min. Later, the fluorescence probe solution was added and followed by 3 rounds of washing with a 0.1% Glucose/PBS solution. Images were obtained using fluorescent microscopy (BZ-9000; KEYENCE, Tokyo, Japan) and quantified using ImageJ software.

### 4.9. Organoid Xenograft Studies

The experiments were carried out following the recommendations of the ‘Guide for the Care and Use of Laboratory Animals and agreed upon by the ethics committees of Tokyo University of Agriculture and Technology (Approval No. R04-120; approval date: 1 June 2022). Male SCID (C.B-17/IcrHsd-Prkdc^scid^) mice were obtained from Japan SLC Inc. (Shizuoka, Japan). A mouse xenograft model of BC 2.5D organoids was performed as described previously [21]. Briefly, 1 × 10^6^ organoid cells were subcutaneously injected into the right flank of mice. After implantation, the longest and shortest diameters of the tumor were measured using a vernier scale, and tumor volumes were estimated using $V=(L\times W^{2})/2$, where L is the longest diameter (length), and W is the shortest diameter (width). After the mean tumor volumes had reached ~100 mm^3^ (day 1), the mice were divided into two groups (n = 10), and JPH203 was administered intraperitoneally five times a week at a dose of 50 mg/kg [35]. JPH203 was dissolved in PBS with 10% Tween^®^ 20. Tumor volumes and body weight were measured twice a week. After the third cycle of JPH203 treatment, mice were anesthetized with isoflurane and the tumors were dissected. The tumor weights were measured and then the tumor tissues were embedded in 4% PFA overnight for paraffin embedding or stored at −20 °C for protein extraction. The paraffin-embedded tissues were further prepared for H&E staining and immunohistochemistry staining. The protein extraction was conducted by CelLytic^TM^ M after grinding, following the method described above in Section 4.6.

### 4.10. Histology of H&E Staining and Immunohistochemical Examination

The H&E staining of xenografted tumor sections was carried out following the standard procedures using paraffin-embedded tissues and images were taken by light microscopy (BX43; Olympus, Tokyo, Japan). The immunohistochemical staining was carried out as described previously [36]. After deparaffinization of tumor tissue sections, antigen retrieval was achieved by a citrated buffer at 121 °C for 5 min, and the endogenous peroxidase activity was terminated by 1% peroxidase for 30 min at RT. Sections were blocked with 10% normal goat serum for 30 min, probed with primary antibodies, and kept at 4 °C overnight. After washing, the sections were treated with Dako Envision+ dual Link System-HRP following the manufacturer’s protocol. The images were taken by light microscopy and quantified using ImageJ software.

### 4.11. Statistical Analysis

Statistical analyses were performed using R software (version 4.4.1). Data are expressed as mean ± standard error of the mean (S.E.M.). Prior to testing, the normality of the data and homogeneity of variances were verified using the Shapiro–Wilk test and F-test, respectively. Variation in Student’s *t*-test were applied based on the experimental design: unpaired *t*-test with Welch’s correction for unequal variances for independent two-group comparisons, and paired *t*-test for paired observations within the same experimental run. For screening across multiple cell lines, a one-way analysis of variance (ANOVA) followed by Tukey’s honestly significant difference test was performed. Where appropriate, *p*-values were adjusted using the Bonferroni correction to account for multiple comparisons. A *p*-value or adjusted *p*-value of less than 0.05 was considered statistically significant.

## 5. Conclusions

**Figure 6:**
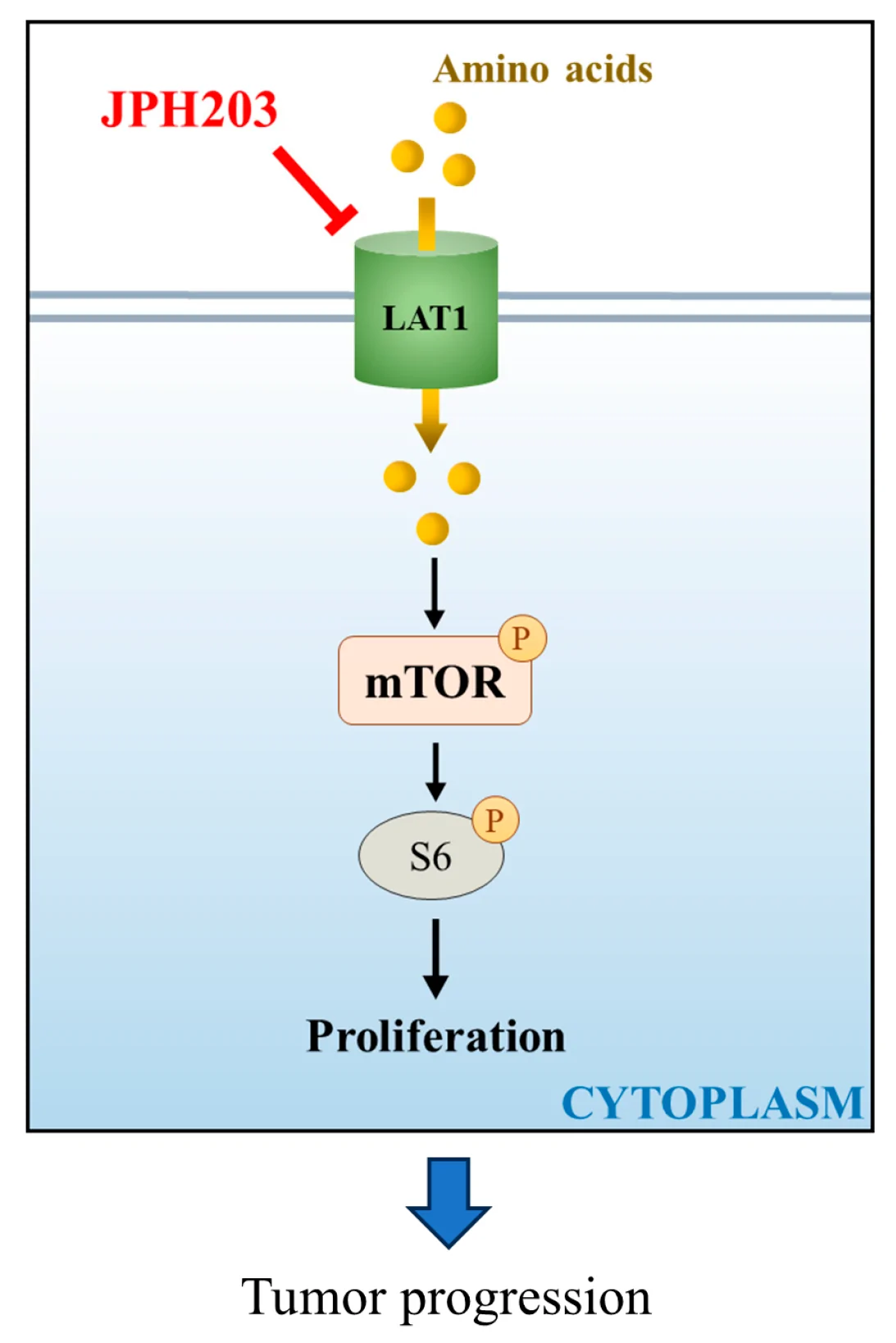
Schematic model of the mTOR signaling pathway and JPH203 therapeutic mechanisms. The red T-shaped line indicates the inhibition of LAT1 by JPH203.

Blockade of LAT1 by JPH203 inhibited canine BC progression and cell proliferation through modulation of the mTOR signaling pathway (Figure 6). Since LAT1 expression has been associated with tumor aggressiveness, our findings suggest that JPH203 may be a promising candidate for further investigation as a LAT1-targeted therapeutic strategy for canine BC. Furthermore, naturally occurring BC in pet dogs shares histopathological features, biological behavior, and therapeutic responses with human BC, supporting the value of this model for the preclinical evaluation of LAT1-targeted therapies in both canine and human BC.

## Figures and Tables

**Table 1:** Sample information.

Case ID	Age (Years Old)	Breed	Sex	Diagnosis
MT1	14	Miniature Dachshund	Female	Mammary adenocarcinoma with lung metastasis
HCC1	11	Chihuahua	Female (spayed)	Hepatocellular carcinoma
KT1	10	Chihuahua	Male	Renal cell carcinoma with liver metastasis
ML1	13	Miniature Dachshund	Male	Oral malignant melanoma (mandibular)
ML2	15	Miniature Dachshund	Female	Oral malignant melanoma
BC1	15	Mixed	Female (spayed)	Urothelial carcinoma with positive BRAF mutation
BC2	16	Yorkshire Terrier	Male (castrated)	Urothelial carcinoma with positive BRAF mutation
BC3	11	Pomeranian	Male	Urothelial carcinoma
BC4	12	Chihuahua	Male	Urothelial carcinoma with positive BRAF mutation

Abbreviations: MT, mammary tumor; HCC, hepatocellular carcinoma; KT, kidney tumor; ML, melanoma; BC, bladder cancer; BRAF, B-Raf proto-oncogene, serine/threonine kinase.

## Data Availability

The raw data supporting the conclusion of this article will be made available by the corresponding authors, upon a reasonable request.

## Supplementary Materials

The following supporting information can be downloaded at: https://www.mdpi.com/article/10.3390/ijms27177681/s1.
