# Supplementary material for: Anti-Tumor Potential of Selective L-Type Amino Acid Transporter (LAT1) Inhibitor, JPH203, in Patient-Derived Canine Bladder Cancer Organoids
Source: Int J Mol Sci. 2026 Aug 27;27(17):7681. doi: 10.3390/ijms27177681 (PMC13566054; doi:10.3390/ijms27177681)
Supplement: Supplementary file 1 [file ijms-27-07681-s001.zip › ijms-4508454-supplementary.pdf]

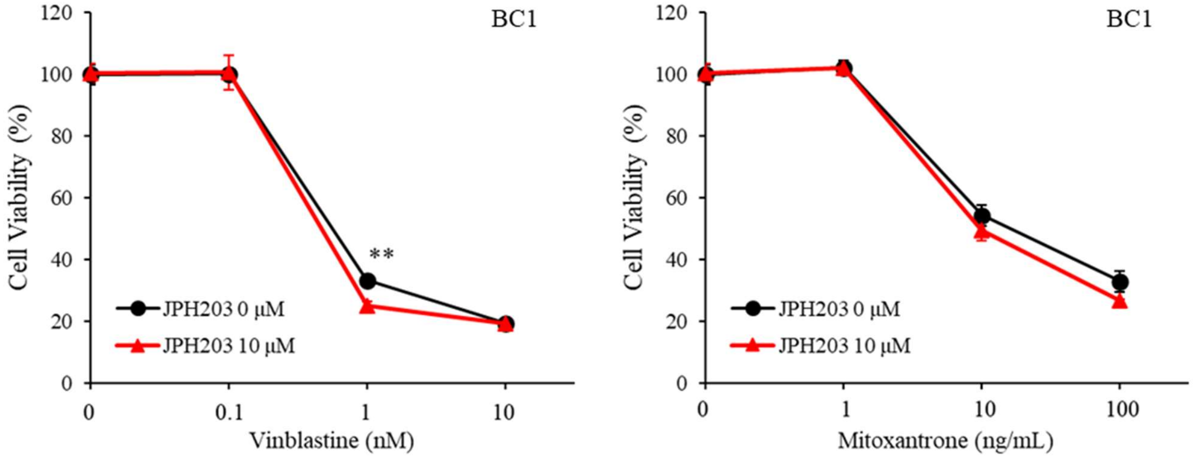

Figure S1. In vitro cell viability of BC1 after vinblastine (0.1–10 nM) or mitoxantrone (1–100 ng/ml) treatment combined with JPH203 (10 µM) for 72 h. ** $p < 0.01$.

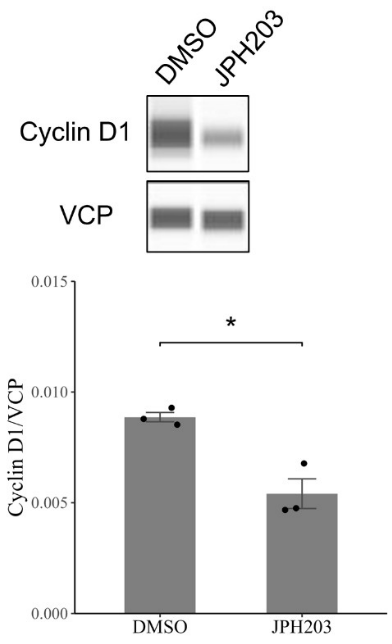

Figure S2. The protein level of Cyclin D1 in BC1 organoids following 24 h JPH203 treatment was determined by Simple Western System (Jess System 004-650A-T001, BioTechne). * $p < 0.05$.

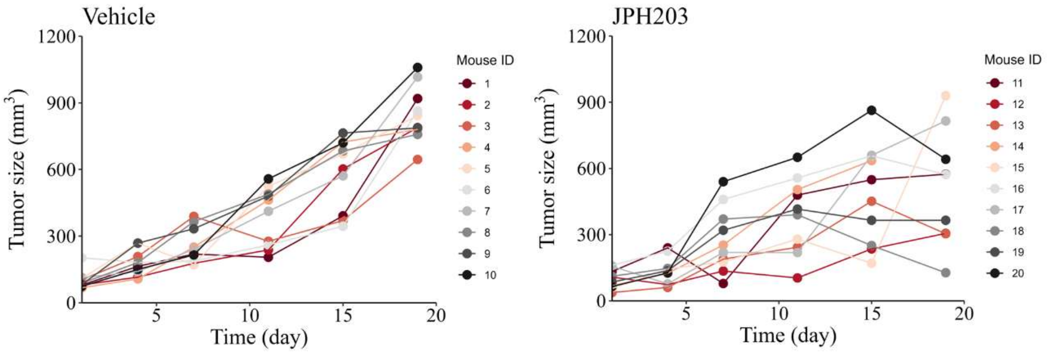

Figure S3. Individual tumor growth curve without (control) or with JPH203 treatment five times a week.

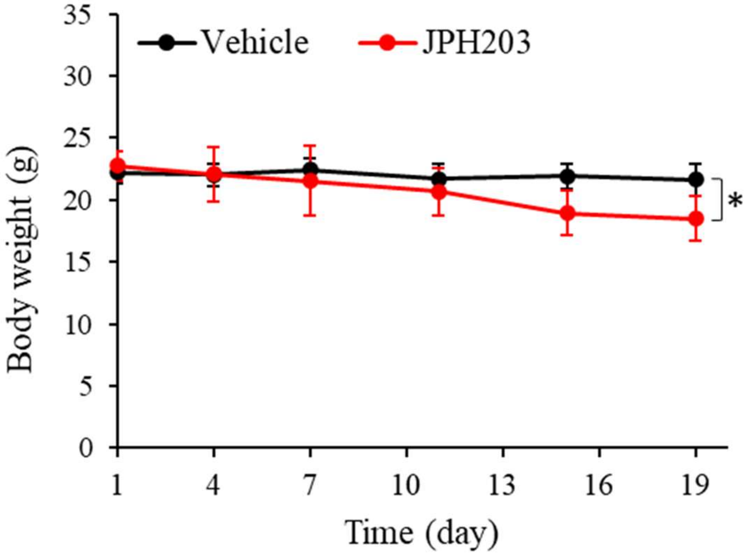

Figure S4. Body weight of mice during 19 days without (control) or with JPH203 treatment five times a week. * $p < 0.05$.
